# Toxin Production and Resistance of *Staphylococcus* Species Isolated from Fermented Artisanal Dairy Products in Benin

**DOI:** 10.1155/2020/7938149

**Published:** 2020-10-28

**Authors:** Majoie Géroxie Tohoyessou, Wassiyath Mousse, Haziz Sina, Fernique Kona, Tania Azanghadji, Nathalie Guessennd, Farid Baba-Moussa, Thomas Dadie, Adolphe Adjanohoun, Lamine Baba-Moussa

**Affiliations:** ^1^Laboratory Biology and Typing Molecular in Microbiology, Faculty of Science and Technology, University of Abomey-Calavi, 05 BP 1604, Cotonou, Benin; ^2^Antibiotics, Natural Substances and Surveillance of Resistance of Microorganisms to Anti-Infective Unit (ASSURMI), Institute Pasteur of Ivory Coast, 01 BP 490, Abidjan 01, Côte d'Ivoire; ^3^Laboratory of Microbiology and Food Technology, Faculty of Science and Technology, University of Abomey-Calavi, ISBA-Champ de Foire, 01 BP 526, Cotonou, Benin; ^4^Laboratory of Biotechnology and Food Microbiology, University Nangui Abrogoua, 02 B.P. 801, Abidjan 02, Côte d'Ivoire; ^5^Benin National Institute of Agricultural Research, Cotonou, Benin

## Abstract

*Staphylococcus* species are considered as one of the major pathogens causing outbreaks of food poisoning. The aim of this work was to assess the toxinogenic and antibiotic susceptibility profiles of the strains of *Staphylococcus* spp isolated from three types of fermented dairy products (yoghourt, millet *dêguê*, and couscous *dêguê*). The isolation of the *Staphylococcus* strains was performed on selective media, and their identification was done using biochemical and molecular methods. The susceptibility at 15 antibiotics tested was assessed using the disc diffusion method. The immunodiffusion method was used to evaluate the toxin (luk-E/D, luk-S/F, ETA, and ETB) production. Biofilm formation was qualitatively researched on microplates. Less than half (42.77%) of the collected samples were contaminated with *Staphylococcus* spp. The yoghourt and millet *dêguê* samples collected in the afternoon were more contaminated than those collected in the morning. The *S. aureus*, *S. capitis,* and *S. xylosus* strains, respectively, were the most present. *S. aureus* was the only coagulase-positive species identified in our samples. The highest resistance to antibiotics was observed with penicillin (100%) irrespective of the nature of the sample. *S. aureus* strains were highly (71.4%) resistant to methicillin. The *S. aureus* strains were the most biofilm-forming (27.6%), followed by *S. capitis* strains. Panton and Valentine's leukocidin (luk-S/F) was produced by only *S. aureus* strains at a rate of 8.33%. Only coagulase-negative *Staphylococcus* (CNS) produced Luk-E/D. The high rates of *Staphylococci* contamination indicate bad hygiene quality during the production and distribution of dairy products. It is, therefore, necessary to improve the quality of fermented milk products.

## 1. Introduction

Milk and its derivative products are very important in children's diets. Fluid milk is a single food providing calcium, potassium, phosphorus, lactose, casein phosphopeptide, and vitamin D in children [1]. Thus, dietary calcium comes mainly from dairy products such as milk, yoghourt, and cheese [2, 3] as well as other dairy products [4]. In West African countries, the most consumed are dèguè (millet and couscous), yoghourt, gappal, and tchobal [5]. Over the last few years, food poisoning and food safety have become very topical subjects, drawing much public concern [6]. Foodborne infections are very common and are related to the consumption of many foods including drinks sold in catering and contaminated with certain bacteria or their toxins. Milk products derived from dairy cows milk can harbor a variety of microorganisms [7] and can be important sources of foodborne pathogen [8]. Various bacteria strains (*Clostridium botulinum, Clostridium perfringens, Campylobacter, Escherichia coli, Salmonella,* and *Staphylococcus aureus*) can cause food poisoning [9]. Among the mentioned bacteria, *Staphylococcus aureus* is a major bacterial pathogen responsible for a broad and divergent range of human and animal infections, including toxin-mediated foodborne diseases [10, 11]. *S. aureus* is reported to be one of the important causes of bovine mastitis and one of the most cost-intensive diseases in the dairy industry [12–14].

To control bacterial infections, the uncontrolled use of antibiotics by self-medication has select resistance bacteria. Unfortunately, most of the antibiotics known to date face bacterial resistance [15]. It was thus reported that methicillin-resistant *S. aureus* (MRSA) can form a powerful biofilm and easily colonize the mucous membranes, which is one of the reasons causing chronic, recurrent, or invasive infections [16]. Their capacity to adhere and form biofilms on the surface of milk processing equipment could contribute to be a source of *S. aureus* contamination of dairy production [17, 18]. Recently, MRSA strains have been detected in various types of food products, including meat products, raw products, milk, and dairy products all over the world [19–21].

Resistance makes the treatment of bacterial infections through conventional antibiotics difficult. In addition, *S. aureus* is often associated with a wide variety of virulence factors such as the production of Panton and Valentine's leukocidin (PVL) and heat-resistant staphylococcal enterotoxins which, when ingested, can cause gastrointestinal disorders [22]. Dairy products generally harbor enterotoxigenic *S. aureus* strains that can induce foodborne intoxications in humans [23, 24]. The produced enterotoxins are reported to cause abdominal cramps, nausea, emesis, and eventually diarrhea [25, 26].

They are increasingly diagnosed in Benin where collective catering, which has become a phenomenon of modern societies due to its nutritional and socioeconomic importance, has considerably increased in recent years [27]. In view of the many cases of food poisoning caused by *S. aureus*, the increased level of methicillin-resistant and toxin producer *Staphylococcus spp* strains from food is to be considered. It is necessary that research be carried out in order to prevent the children health risks linked to the consumption of fermented dairy products. The aim of this study was to assess the toxinogenic and antibiotic susceptibility profiles of *Staphylococci* strains isolated from three types of fermented dairy products (yoghourt, couscous *dêguê*, and millet *dêguê*) sold in secondary schools in Cotonou and Abomey-Calavi in Benin.

## 2. Materials and Methods

### 2.1. Sampling and Sample Collection

In this study, 15 schools in Cotonou and Abomey-Calavi were selected using the “purposive” sampling technique for the sample collections [28]. Using this sampling method, 180 samples of the three selected fermented milk products (yoghourt, millet *dêguê,* and couscous *dêguê*) were collected from vendors located inside the schools or outside within a radius of 20 meters. The collected samples include 60 yoghourt samples, 60 millet *dêguê* samples, and 60 couscous *dêguê* samples. For each type of fermented dairy products, two samples were taken twice a day (morning and evening) and twice per week. Once collected, the samples were transported into an icebox (about 4°C) to the laboratory for microbial analysis.

### 2.2. Microbiological Analyses

#### 2.2.1. Isolation and Identification of *Staphylococcus spp*

*Staphylococcus* strains were isolated on Baird–Parker agar (OXOID CM0275) enriched with egg yolk and potassium tellurite [29]. Briefly, 10 g of each collected sample was homogenized into a sterile bottle, a stomacher with 90 ml of sterile tryptone salt water. After incubation at 37°C for 24 hours, the isolated colonies were characterized by biochemical tests (catalase test, coagulase test, DNase test, and gallery API®STAPH) [30].

#### 2.2.2. Molecular Confirmation of *Staphylococcus spp*

The isolated *Staphylococcus* strains were confirmed molecularly through the PCR. The total DNA was manually extracted using the boiling method [31]. The PCR was performed in 30 *µ*l containing 15 *µ*l of 2x Master Mix (Biolabs), 1.5 *µ*l of forward primer (G1: 5′-GAAGTCGTAACAAGG-3′), 1.5 *µ*l of reverse primer (L1: 5′-CAAGGCATCCACCGT-3′), and 3 *µ*l of DNA. 25 cycles (94°C for 1 minute, 50°C for 30 s, and of 72°C for 1 min) was performed in a thermocycler (MultiGene, Labnet International, Inc.). The initial denaturation was done at 94°C for 5 min, the final elongation was done at 72°C for 7 min, and the amplified product was stored at 4°C until electrophoresis migration. The electrophoresis was performed at 150 V for 30 min on a 1.5% agarose gel containing ethidium bromide. A 100 bp standard molecular ladder was used. The bands of 16 S–23 S gene of *Staphylococcus spp* were visualized at approximately 437 bp [32] on the UV transillumination.

### 2.3. Phenotypic Detection of Toxins

The production of Panton and Valentine's leucocidin (luk-S/F), leukotoxin Luk-E/D, and the epidermolysins (ETA and ETB) was investigated on isolated strains by the radial immunoprecipitation method [33]. Briefly, fresh *Staphylococcus* colonies were cultured in 500 *µ*l of yeast casamino acid-pyruvate (YCP) broth at 37°C for 18–24 h with stirring (200 rpm). The supernatants of each sample of bacterial culture are collected after centrifugation (5,000 rpm for 5 min). On a 0.6% agarose gel prepared with PBS, seven wells spaced 8 mm were dug. About 30 *µ*l of each supernatant was deposited in the corresponding external well. The appropriate purified rabbit antibodies (OD = 3) are deposited in the central rosette well and control antigens (OD = 0.2) in the top and bottom wells. The experiment was incubated at room temperature for about 16 hours. After incubation time, the precipitation arcs were observed directly or after staining with Coomassie blue [33].

### 2.4. Biofilm Training Research

From an 18 h culture in brain heart infusion, 48-well microplates (polystyrene) were inoculated with 10 *µ*l of dilute bacteria suspension to which 150 *µ*l of BCC was added. The microplates were incubated for 24 h at 37°C. After incubation, the wells were washed three times with sterile physiological water (about 0.2 ml) to remove free bacteria (plankton). The biofilms formed by the adhesion of sessile organisms to the polystyrene support in each of the wells were stained with crystal violet (0.1%) for 10 min. The excess dye was thoroughly removed with sterile distilled water, and the plates were left at room temperature for drying. The results were compared to the positive and negative controls after incubation [34].

### 2.5. Antibiotics Susceptibility of Isolated Strains of *Staphylococcus spp*

The susceptibility of each *Staphylococcus* strain to antibiotics was determined by the diffusion method [35]. The tested antibiotics (Bio-Rad) were as follows: penicillin G (P 10 *µ*g), amikacin (AK 30 *µ*g), fosfomycin (FOS 50 *µ*g), cefoxitin (FOX 30 *µ*g), gentamycin (GEN 10 *µ*g), erythromycin (E 15 *µ*g), lincomycin (MY 15 *µ*g), ciprofloxacin (CF 5 *µ*g), ofloxacin (OFX 5 *µ*g), amoxicillin (AMO 20 *µ*g), cefotaxime (CTX 30 *µ*g), tetracycline (TET 30 *µ*g), trimethoprim-sulfamethoxazole (SXT 23.75 *µ*g), amoxicillin-clavulanic acid (AMC 20 *µ*g), and fusidic acid (FD 10 *µ*g).

### 2.6. Data Analysis

Microsoft Office Excel 2010 spreadsheet was used for data processing. The statistical analysis and graphs were made using the R 3.6.1. The test is considered statistically significant if *p* < 0.05.

## 3. Results

### 3.1. Microbiological Quality of the Collected Fermented Dairy Products

#### 3.1.1. Enumeration of *Staphylococcus spp* Germs in the Fermented Dairy Products Analyzed

The results of germ enumeration in fermented dairy products collected, expressed in CFU/ml, are presented in Table 1. They represent the microbial load of the various microorganisms sought in the yoghourt, the millet *dêguê,* and the couscous *dêguê* analyzed. The analysis of this table shows that the microbial loads vary according to sample types. The most contaminated samples were those of millet *dêguê* and the least contaminated were those of yoghourt (754.3*∗*10^3^ CFU/ml). Regarding total coliforms, millet *dêguê* samples were the most contaminated (1166.7*∗*10^3^ CFU/ml) and the least contaminated were the yoghourt samples.

#### 3.1.2. *Staphylococcus* Species Found in the Fermented Dairy Products Analyzed

**Figure 1:**
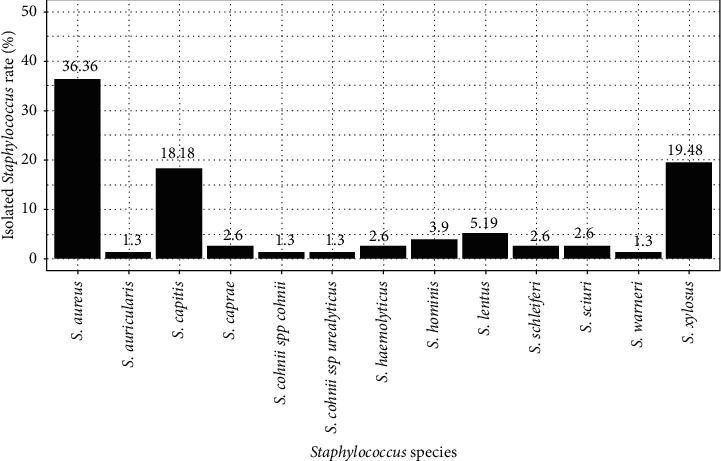
Rate of the different species of *Staphylococcus* found.

*Staphylococcus spp* strains were isolated at 42.77% from the 180 collected samples. The 16S–23S gene from *Staphylococcus spp* was present in all of our isolated strains. A total of 13 species of *Staphylococcus* were identified. *S. aureus* was the only coagulase-positive species identified in our samples. The three most represented species were *S. aureus* (36.36%), *S. xylosus* (19.48%), and *S. capitis* (18.18%). *S. auricularis*, *S. cohnii spp cohnii*, *S. cohnii spp urealyticus,* and *S. warneri* were the least isolated with a respective rate of 1.3% (Figure 1).

#### 3.1.3. Distribution of *Staphylococcus spp* Strains Isolated according to the Fermented Dairy Products

**Figure 2:**
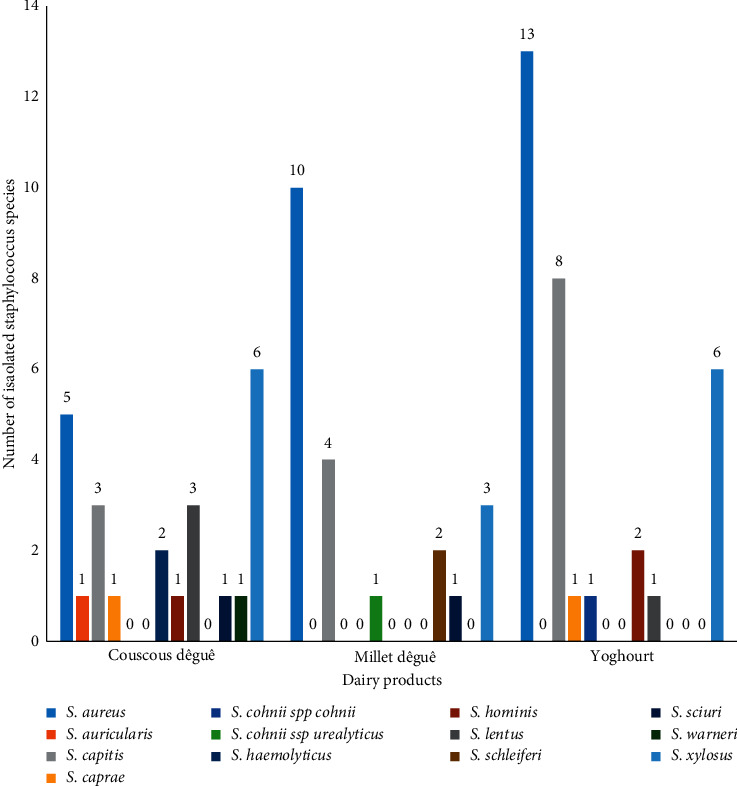
Distribution of *Staphylococcus spp* strains according to fermented milk products.

Figure 2 shows the distribution of the 13 identified *Staphylococcus* strains according to the kind of fermented milk products. The distribution of species according to the different types of fermented milk products is not statistically significant (*p* > 0.05). However, it appears that *S. aureus* was the most isolated irrespective of the milk products. Ten different species were identified in the couscous *dêguê*, seven species in yoghourt, and six species in the millet *dêguê*. *S. aureus*, *S. xylosus,* and *S. capitis* were present in three kinds of products.

#### 3.1.4. Distribution of *Staphylococcus spp* Strains Isolated according to the Time of Collection

**Figure 3:**
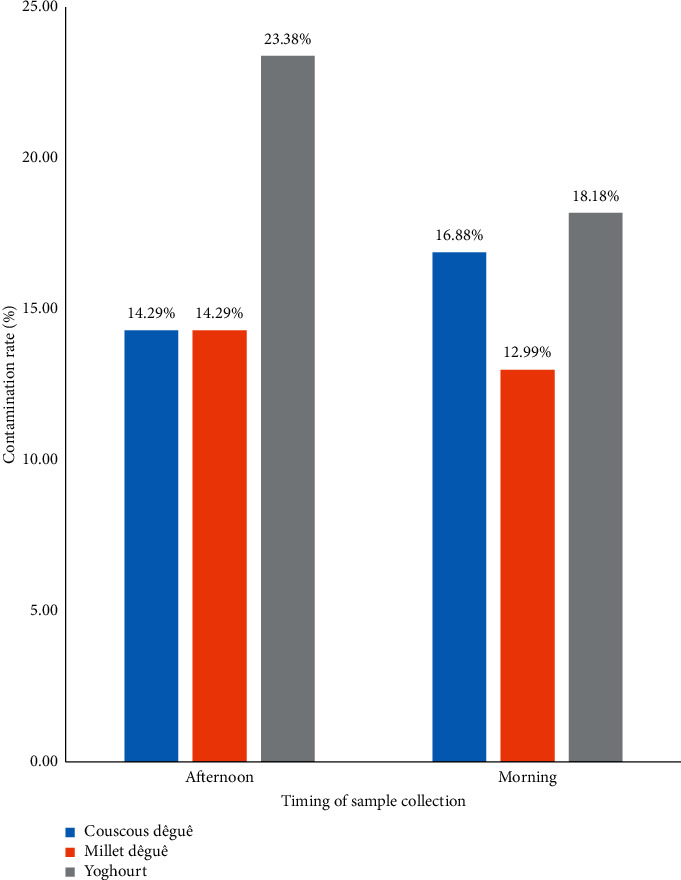
Distribution of *Staphylococcus spp* strains according to the time of collection.

Figure 3 shows that couscous *dêguê* samples were more contaminated in the morning (16.29%) than in the afternoon (14.29%). The yoghourt samples were more contaminated in the afternoon (23.38%) than in the morning (18.18%).

### 3.2. Distribution of Biofilm Formation according to the Isolated *Staphylococcus* Species

**Figure 4:**
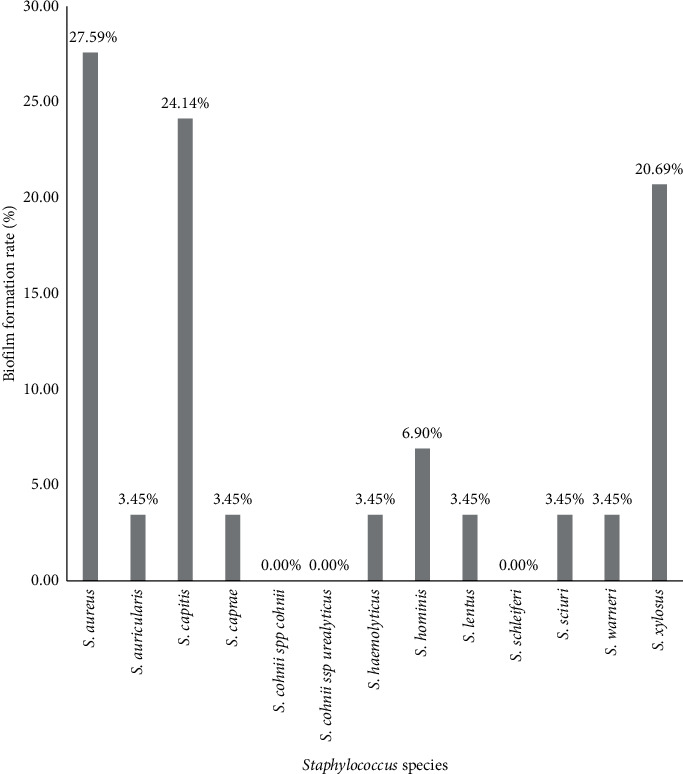
Biofilm production rate by isolated *Staphylococcus* species.

Biofilm production does not vary statistically according to the *Staphylococcus* species (*p* > 0.05). The biofilm production capacity of isolated *Staphylococcus* shows that *S. aureus* was the most biofilm (27.6%) formative followed by *S. capitis* (24.1%) and *S. xylosus* (20.7%) (Figure 4). None of *S. cohnii spp cohnii*, *S. cohnii ssp urealyticus,* and *S. schleiferi* produced biofilm.

**Figure 5:**
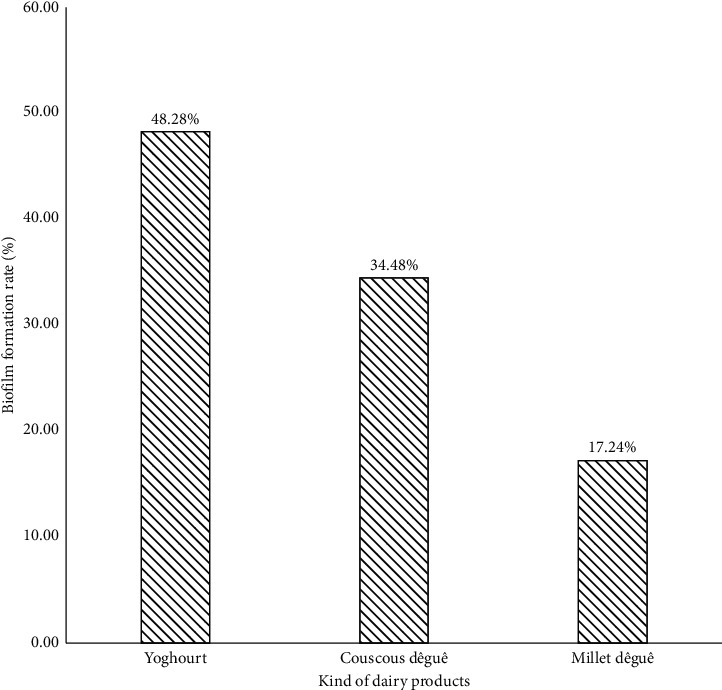
Biofilm production rate according to *Staphylococcus spp* strains in the different samples.

The *Staphylococcus* strains isolated from yoghourt (48.28%) made more biofilm while those isolated from couscous *dêguê* (17.24%) made less (Figure 5). Biofilm production by the different isolated species in the function of sample types is statistically significant (*p*=0.042).

### 3.3. Distribution of Toxin Production by Isolated *Staphylococcus spp* Strains

**Figure 6:**
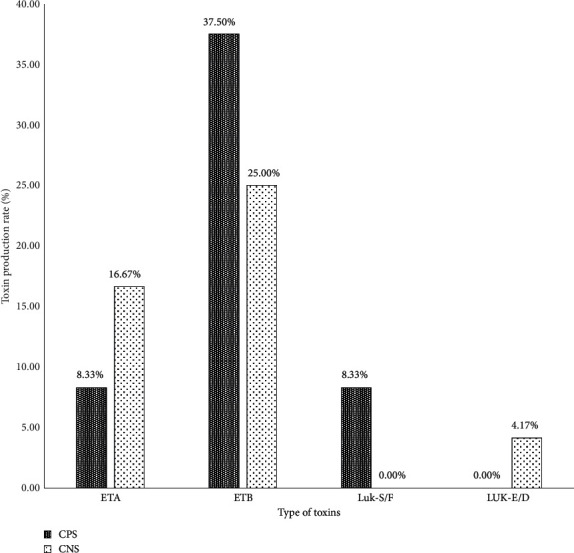
Rate of toxin production by *Staphylococcus spp* strains.

The isolated *Staphylococcus* spp were predominantly producing epidermolysin B (ETB) at a rate of 37.5% for coagulase-negative *Staphylococcus* strains and 25% for coagulase-positive *Staphylococcus*. Luk-S/F was produced by only *S. aureus* strains at a rate of 8.33%. Only coagulase-negative *Staphylococcus* have produced Luk-E/D (Figure 6).

**Figure 7:**
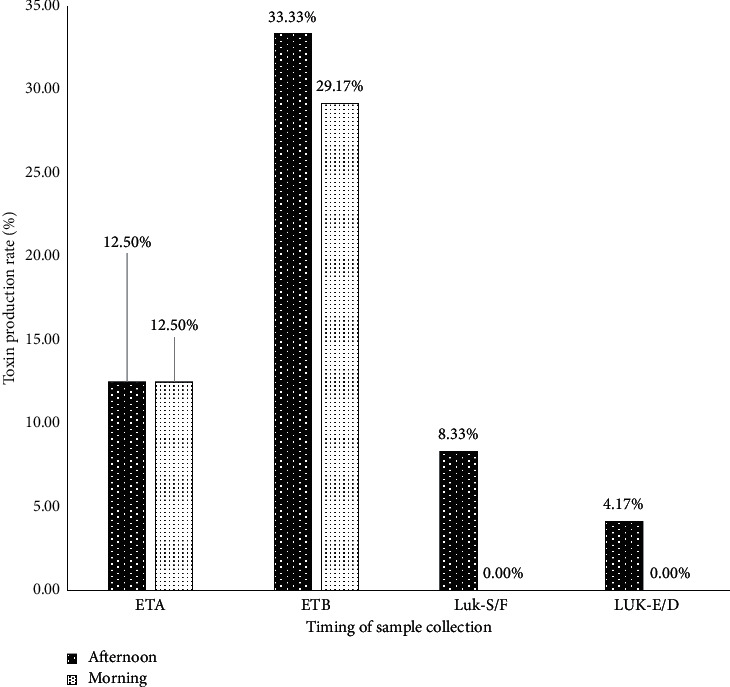
Rate of toxin production by *Staphylococcus* spp according to the timing of sample collection.

In the samples collected in the morning, only ETA and ETB toxins were produced by the *Staphylococcus* strains. ETB-producer strains were present in the afternoon samples. Luk-E/D and Luk-S/F toxins were produced only by *Staphylococcus* strains isolated in the afternoon (Figure 7).

### 3.4. Susceptibility of Isolated *Staphylococcus spp* Strains to Antibiotics

**Figure 8:**
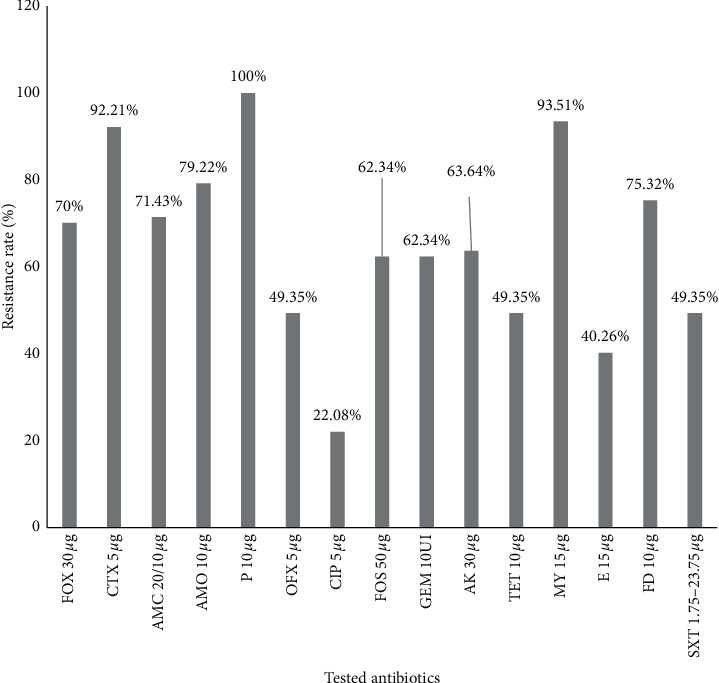
Resistance rate of isolated *Staphylococci* strains to antibiotics.

All the *Staphylococcus* strains were resistant to penicillin, and 93.51% were resistant to lincomycin. About 70.13% were resistant to cefoxitin, and 60% were resistant to the antibiotics of the *β*-lactam family. The lowest resistance rate was observed with ciprofloxacin (22.08%) (Figure 8).

**Figure 9:**
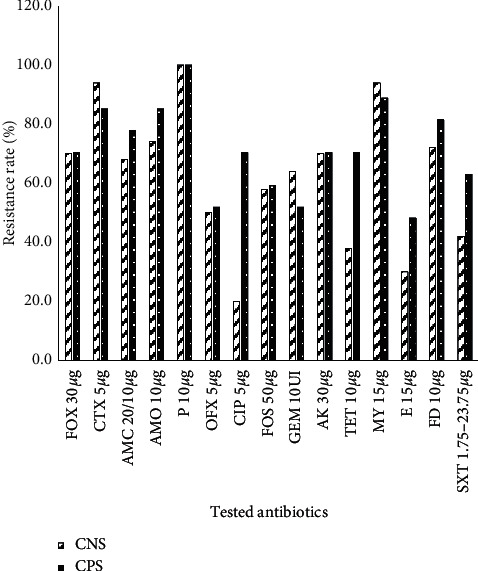
Antibiotic resistance rate of strains of negative coagulase *Staphylococcus spp* (NCS) and *S. aureus* (CPS) isolated.

For coagulase-negative *Staphylococcus* (CNS), the resistance of 6 antibiotics (ciprofloxacin, erythromycin, ofloxacin, trimethoprim-sulfamide, and tetracycline) was less to 50%. The lowest resistance was observed on *S. aureus* with ciprofloxacin (28.6%), followed by erythromycin (39.3%). The resistance of *S. aureus* to methicillin (cefoxitin) was 71.4% (Figure 9).

## 4. Discussion

The evaluation of the microbiological quality of fermented milk (yoghourt, millet *dêguê*, and couscous *dêguê*) products reveals the presence of total coliforms and *Staphylococcus* germs. This observation is consistent with the work of Kukhtyn et al. [36], who find that the greatest danger in the process of making milk and milk products is the contamination of pathogens. Dairy products are highly moisture and offer suitable growth conditions for many pathogens including *S. aureus* that has frequently been the cause of foodborne diseases [37]. It is thus important to implement microbiological regulations for each dairy product to reduce food poisoning by fermented dairy products. The presence of *S. aureus* illustrates a failure in hygiene and in the implementation of good manufacturing practice. The search for these germs at the industrial level constitutes a test of overall hygienic quality. The microbiological quality of dairy products is important in the prevention of food poisoning. Thus, in this study, we observed a very high presence of total coliforms and *Staphylococci*. It emerged from our investigations that 36.36% of the strains of *Staphylococci* isolated were coagulase-positive (*Staphylococcus aureus*) and 63.64% were coagulase-negative (Figure 1). The presence of *S. aureus* in dairy products could alter the microbiological quality of these products and cause food poisoning [38]. Worldwide, *S. aureus* has been isolated from several ready-to-eat products. The presence of those microorganisms indicates potentially cross-contamination that may be due to improper staff hygiene and/or poor surface sanitation. Indeed, *S. aureus* strains were isolated from food handlers, foods, and patient specimens [39]. This microorganism is capable of surviving on dry stainless steel, and it can easily be transferred from sponges to stainless steel surfaces and subsequently to food products [40]. In milk transformation, *S. aureus* can contaminate the production at almost every step. Therefore, *S. aureus* can be shed directly into the milk and produce enterotoxins that represents one of the most common food safety concerns from raw milk products [41–43].

As in our study, some authors observed in the United States [44], Egypt [45], and Morocco [46] diversity in the presence of coagulase-negative *Staphylococcus* strains. The ones commonly detected in raw milk are *S. epidermidis*, *S. simulans*, *S. hominis*, *S. caprae*, *S. warneri,* and *S. xylosus* [44, 47, 48]. In our study, the most dominant coagulase-negative strain was *S. xylosus*. But Organji et al. [48] observed in their study on milk in Egypt that *S. saprophyticus* was the most dominant coagulase-negative strain. This difference in proportion can be explained by the fact that our samples were not fermented enough because *S. saprophyticus* plays a role in the fermentation process [49]. In this respect, probably no health risks are associated with all *Staphylococci* detected in fermented dairy products samples. Considering the collection period, the yogurt samples are most contaminated by *Staphylococci* in the afternoon (23.38%) than in the morning (18.18%). This result is lower than the results found by Tondo et al. [50] who found 90.4% contamination of *S. aureus* in raw milk taken in the morning. This difference could be explained by the fermentation and pasteurization processes that yogurt undergoes.

Concerning the formation of biofilm capability, *S. aureus* displays the highest rate (27.6%). In addition, the yogurt samples were those that contained more strains (48.28%) of biofilm-forming *Staphylococci*. Indeed, *S. aureus* can produce a multilayer biofilm incorporated into a glycocalyx with the expression of heterogeneous proteins overall, forming at least two types of biofilms: ica-dependent and ica-independent [51]. The formation of biofilm by foodborne staphylococcal strains (especially yogurt) is very serious for human prognosis, especially for children who are the biggest consumers of it. This biofilm formation is more observed in clinical strains [52]. Biofilm formation in dairy equipment as well as insufficient acidification during fermentation opens *S. aureus* niches for multiplication and efficient contamination of the dairy processing lines [17].

The pathogenicity of *Staphylococcus* strains is attributable to toxin production and their antibiotic-resistant profile [53]. Toxin research revealed that Panton and Valentine's leucocidin was produced (8.33%) only by *S. aureus*. The most produced toxin is epidermolysin B (62.5%) by all strains of *Staphylococcus*. This result is higher than the 27.59% founded by Ahouandjinou et al. [54] among the *Staphylococci* strains isolated from bovine carcasses in Benin. The PVL production obtained in our study is lower than the 15% obtained in Benin by Baba-Moussa et al. [55] for direct debits of all origins. Baba-Moussa et al. [56] showed that 21.50% of the *S. aureus* strains isolated from CHU infections produced PVL. This toxin, in clinical practice, is associated with skin diseases such as boils and abscesses [57]. Thus, the production of PVL by food strains must also challenge us especially in terms of its virulence. This presence may be due to the carrying of these types of ailments by sellers that would facilitate the transmission from humans to food. *S. aureus* is able to survive on dry stainless steel and it can easily be transferred from any surfaces to food products [40]. Therefore, in the milk transformation process, *S. aureus* can contaminate the production at almost every step. *S. aureus* can be shed directly into the milk and produce exotoxins that represents one of the most common food safety concerns from raw milk products [41–43, 58]. In addition, toxins can be produced in the product during storage of the fermented dairy product if conditions allow the growth of *S. aureus*.

The study of the sensitivity to antibiotics of isolated *Staphylococcus* strains showed the existence of resistance, with variable proportions according to the families of antibiotics. In fact, the *Staphylococcus* spp show a high resistance rate to penicillin (100%), lincomycin (93.51%), and cefotaxime (90.91%). These results are consistent with those reported on staphylococcal strains isolated from street foods in Benin [10] and Ethiopia [59]. This high resistance level to antibiotics observed can be due to self-medication and excessive and uncontrolled use of antibiotics. The lowest resistance of *S. aureus* observed was with ciprofloxacin (28.6%). This result is close to the 31.3% obtained in studies carried out on milk by Wang et al. [60]. About 71% of the *S. aureus* were resistant to methicillin (cefoxitin). These results are higher than the 15.18% found by Sina et al. [10]. The coagulase-negative staphylococcal strains displayed methicillin resistance rates of 69.4%. Our results concerning this antibiotic are slightly lower than those reported for clinical strains in Turkey on coagulase-negative *Staphylococci* [61]. This difference may be caused by the fact that clinical strains are more in contact with the antimicrobial molecule than food strains. The *Staphylococcus* spp. methicillin resistance observed in this study suggests that it involves the resistance of most of the *β*-lactams currently available [62]. However, the proportion is frightening for food since it is reported that methicillin-resistant *Staphylococcus* strains began to develop resistance to many antibiotics (quinolones, macrolides, aminoglycosides, tetracyclines, trimethoprim-sulfamethoxazole, clindamycin, and chloramphenicol) widely used to control staphylococcal infection [63, 64], such as food poisoning. The sellers who do not protect their hands and head and do not observe hygiene rules during the manufacturing of dairy products quickly find themselves contaminated by potentially pathogenic *Staphylococcus* strains that are antibiotics-resistant, producing toxins, and forming biofilms. This situation is a real problem for food safety.

## 5. Conclusion

Staphylococcal food poisoning is of major concern in public health programs worldwide. Results clearly indicated that the fermented milk products analyzed were contaminated with 13 species of *Staphylococcus,* namely, *S. aureus.* Human, animal, and environmental sources could be incriminated in the contamination of dairy products. Resistant and toxin-producing strains were also isolated from the collected fermented milk products. There is a high risk of food poisoning associated with the consumption of those dairy products. We can say that good hygienic and manufacturing practices along each step of the selected dairy fermented production chain are of great importance and necessary to eliminate the dissemination of potentially pathogenic *Staphylococci* in the community. Thus, more measures focusing on hygienic prevention are required to reduce contamination by *Staphylococci*.

## Figures and Tables

**Table 1:** *Staphylococcus spp* bacterial load of the samples collected.

Samples	* Staphylococcus spp* (UFC/ml)	Total coliforms (CFU/ml)
Yoghourt	754.3*∗*10^3^	116.3*∗*10^3^
Millet *dêguê*	1166.7*∗*10^3^	1221.9*∗*10^3^
Couscous *dêguê*	772.6*∗*10^3^	769.7*∗*10^3^

## Data Availability

The data are available from the corresponding author upon request.
